# Neutrophilic Skin Lesions in Autoimmune Connective Tissue Diseases: Nine Cases and a Literature Review

**DOI:** 10.1097/MD.0000000000000346

**Published:** 2014-12-02

**Authors:** Estelle Hau, Marie-Dominique Vignon Pennamen, Maxime Battistella, Anne Saussine, Maud Bergis, Benedicte Cavelier-Balloy, Michel Janier, Florence Cordoliani, Martine Bagot, Michel Rybojad, Jean-David Bouaziz

**Affiliations:** From the Dermatology Department (EH, AS, MJ, FC, MarB, MR, JDB) and Pathology Department (MDVP, MaxB, BCB), Paris Diderot University, Sorbonne Paris Cité, AP-HP, Saint Louis Hospital, Paris, France

## Abstract

The pathophysiology of neutrophilic dermatoses (NDs) and autoimmune connective tissue diseases (AICTDs) is incompletely understood. The association between NDs and AICTDs is rare; recently, however, a distinctive subset of cutaneous lupus erythematosus (LE, the prototypical AICTD) with neutrophilic histological features has been proposed to be included in the spectrum of lupus. The aim of our study was to test the validity of such a classification. We conducted a monocentric retrospective study of 7028 AICTDs patients. Among these 7028 patients, a skin biopsy was performed in 932 cases with mainly neutrophilic infiltrate on histology in 9 cases. Combining our 9 cases and an exhaustive literature review, pyoderma gangrenosum, Sweet syndrome (n = 49), Sweet-like ND (n = 13), neutrophilic urticarial dermatosis (n = 6), palisaded neutrophilic granulomatous dermatitis (n = 12), and histiocytoid neutrophilic dermatitis (n = 2) were likely to occur both in AICTDs and autoinflammatory diseases. Other NDs were specifically encountered in AICTDs: bullous LE (n = 71), amicrobial pustulosis of the folds (n = 28), autoimmunity-related ND (n = 24), ND resembling erythema gyratum repens (n = 1), and neutrophilic annular erythema (n = 1). The improvement of AICTDS neutrophilic lesions under neutrophil targeting therapy suggests possible common physiopathological pathways between NDs and AICTDs.

## INTRODUCTION

Neutrophilic dermatoses (NDs) are a group of disorders characterized by skin lesions for which histological examination shows intense inflammatory infiltrate, composed primarily of neutrophils, with no evidence of infection. Classical NDs include Sweet syndrome, pyoderma gangrenosum, subcorneal pustular dermatosis, erythema elevatum diutinum, and other transitional forms. NDs may be associated with a variety of systemic disorders, including myeloproliferative disorders, monoclonal gammopathies (mainly IgA type), inflammatory bowel diseases, and autoimmune connective tissue diseases (AICTDs). At present, the pathophysiology of NDs is poorly understood, but the current knowledge of NDs suggests that they should be categorized within the spectrum of “polygenic” autoinflammatory diseases.^1,2^ One of the prototype polygenic autoinflammatory diseases is inflammatory bowel disease. The exact term “autoinflammatory disease” encompasses an enlarging group of inflammatory disorders, defined as Mendelian genetic diseases (monogenic diseases) of the innate immune system that involve mutations in molecular platforms called “inflammasomes.” This results in an excessive inflammatory cytokine production by the innate immune cells (in particular, interleukin (IL)-1) in response to danger signals. Monogenic autoinflammatory diseases are also characterized by a clinical and biological inflammatory syndrome in which there is little or no evidence of autoimmunity.^3^ One of the prototypic monogenic autoinflammatory diseases is the cryopyrin-associated periodic syndrome, which is caused by NLRP3 selective gene mutations.^4^ NDs share many clinical features of monogenic inflammatory disorders, including fever, arthralgia, and neutrophilic infiltration of the skin and visceral organs.

Connective tissue diseases (CTDs) are a group of disorders that are characterized by abnormal structure or function of one or more of the elements of connective tissues.^5^ AICTDs include lupus erythematosus (LE), dermatomyositis (DM), Sjögren syndrome, rheumatoid arthritis, and systemic sclerosis. The pathophysiological hallmark of AICTDs is the activation of the adaptive immune system against “self” antigens, resulting in the detection of autoantibodies (autoAbs) (produced by plasmocytes, the most mature state of B cells) or self-antigen-specific T cells. Skin lesions in AICTDs, especially in LE,^6^ are generally separated into 2 groups, based on a careful morphological evaluation, evolution, and histological results:

1. Specific skin lesions, which result from autoreactive T lymphocyte infiltrate (sometimes mixed with histiocytes) of the dermis and the basement membrane and/or autoAbs deposition; this is classically associated with vacuolar degeneration of the basal cell layer of the epidermis and apoptotic keratinocytes (interface dermatitis), as is found in LE and DM, but not Sjögren syndrome.
2. Nonspecific CTD skin lesions, which result from vasculitis, thrombosis, or other mechanisms and may be encountered in other disease settings.

For example, acute, subacute, and chronic cutaneous LE, including discoid LE^7^ are LE-specific skin lesions; Raynaud phenomenon, purpura, urticarial vasculitis, livedo, and calcinosis cutis are nonspecific LE skin lesions.

Autoinflammatory and autoimmune diseases share clinical (fever, skin rash, and arthralgia) and biological (systemic inflammation) characteristics, but the resulting inflammation is mainly due to the activation of the innate immune system in autoinflammation (neutrophils) and the adaptive immune system in autoimmunity (lymphocytes).^8^ Associations between AICTDs and neutrophilic infiltration have been reported as pyoderma gangrenosum and LE;^9^ Sweet syndrome and LE,^10^ rheumatoid arthritis,^11^ or DM;^12^ Sweet-like ND and LE;^13^ neutrophilic urticarial dermatosis and LE;^14^ nonbullous histiocytoid neutrophilic dermatitis and LE;^15^ palisaded neutrophilic granulomatous dermatitis and LE;^16^ bullous LE;^17^ ND in systemic lupus erythematosus (SLE), resembling erythema gyratum repens;^18^ amicrobial pustulosis of folds and LE;^19^ autoimmunity-related ND,^20^ also coined by other authors “nonbullous neutrophilic lupus erythematosus”^21^ (nonbullous clinical lesions with interface dermatitis and an unusual neutrophilic infiltrate on histological examination). Notably, in 2010, Lipsker^22^ proposed to include neutrophilic skin lesions in the spectrum of LE skin lesions. The aim of our study was to test the reproducibility and the applicability of this classification in lupus and other AICTDs. Therefore, we describe the clinical and histological spectrum of neutrophilic skin lesions associated with AICTDs through various case reports and through an exhaustive review of the cases that have been published in the literature.

## PATIENTS AND METHODS

**FIGURE 1:**
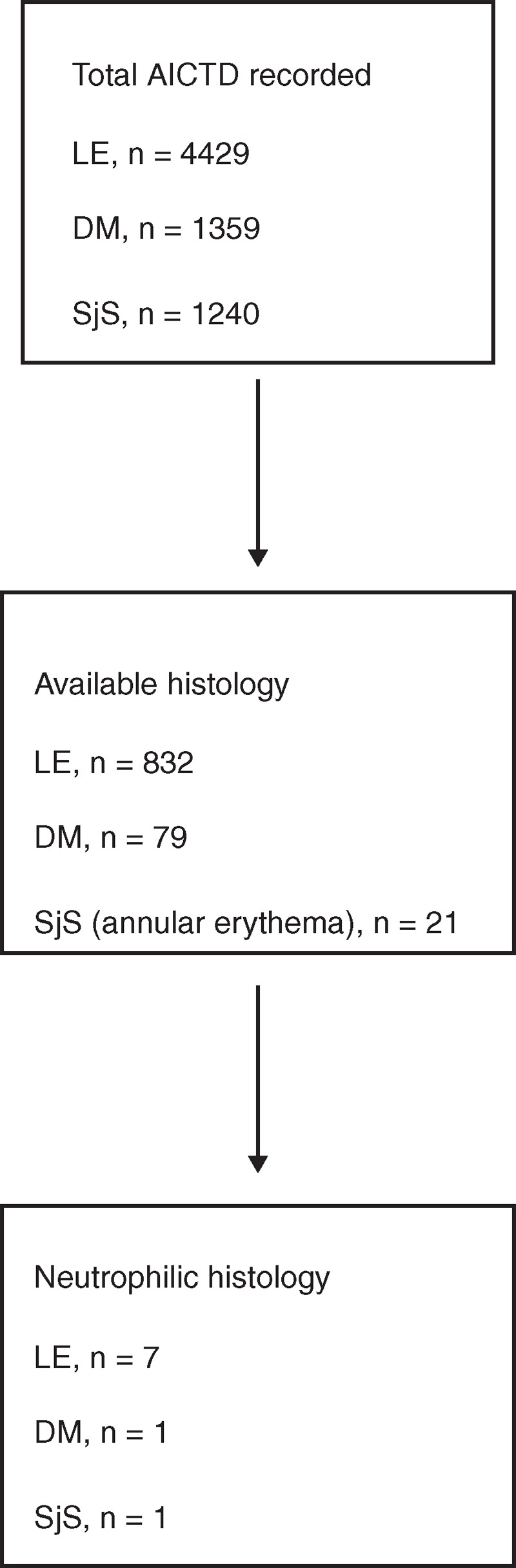
Flow diagram of patients with AICTD neutrophilic skin lesions included in the study. AICTD = autoimmune connective tissue disease, DM = dermatomyositis, LE = lupus erythematosus, SjS = Sjögren syndrome.

We retrieved the medical records of all AICTDs patients for which skin biopsy showed significant neutrophilic infiltrate; records were examined from Saint Louis Hospital (Paris, France), between 2003 and 2013. Among 7028 AICTDs patients, a skin biopsy was available in 932 cases; there was mainly neutrophilic infiltrate on histology in 9 cases (Figure 1).

Patients were included if they met the following criteria:

1. Diagnosis of AICTD, according to the standard criteria of AICTDs, which is based on the revised 1997 American College of Rheumatology (ACR) criteria for SLE.^23^ The ACR 2012 criteria was used for Sjögren syndrome;^24^ the Bohan and Peter^25^ 1975 criteria was used for DM. Cutaneous LE diagnosis was based on clinical criteria of discoid LE (red, scaly patches of variable size, which heal with atrophy, scarring, and pigmentary changes), subacute LE (erythematous macules or papules of sun-exposed areas that evolve into scaly, papulosquamous or annular/polycyclic plaques) or LE tumidus (indurated, succulent, urticaria-like, single or multiple plaques with a bright reddish or violaceous smooth surface without clinically visible epidermal involvement on sun-exposed areas, which is exacerbated during the summer), and/or histological characteristic of LE (interface dermatitis with vacuolar alteration of the basal cell layer of the epidermis and patchy dermal lymphocytic infiltrate, possibly associated with epidermal atrophy and hyperkeratosis)^6^ without systemic involvement.
2. Histological features of neutrophilic skin lesions, including every skin lesion with a significant neutrophilic infiltrate (>50%). Neutrophilic leukocytoclastic vasculitis skin lesions were not included to focus on neutrophilic skin infiltrates that may be related to AICTDs.

Biopsies, medical records, and photographs of patients included were reviewed and analyzed. We also searched the National Library of Medicine's MEDLINE database (Bethesda, MD) for relevant literature using the keywords “neutrophil,” “neutrophilic dermatosis,” “ Sweet syndrome,” “neutrophilic urticaria,” “pyoderma gangrenosum,” “annular erythema,” “palisaded neutrophilic granulomatous dermatitis,” or “amicrobial pustulosis of the folds,” together with “connective tissue disease,” “discoid lupus erythematosus,” “systemic lupus erythematosus,” “Sjogren syndrome,” “rheumatoid arthritis,” and “dermatomyositis.” The bibliographies of all the selected articles were reviewed for additional case reports. Together, 89 different articles, published between 1978 and 2014 in the international literature, were included in this review.

## RESULTS, DISCUSSION, AND LITERATURE REVIEW

**FIGURE 2:**
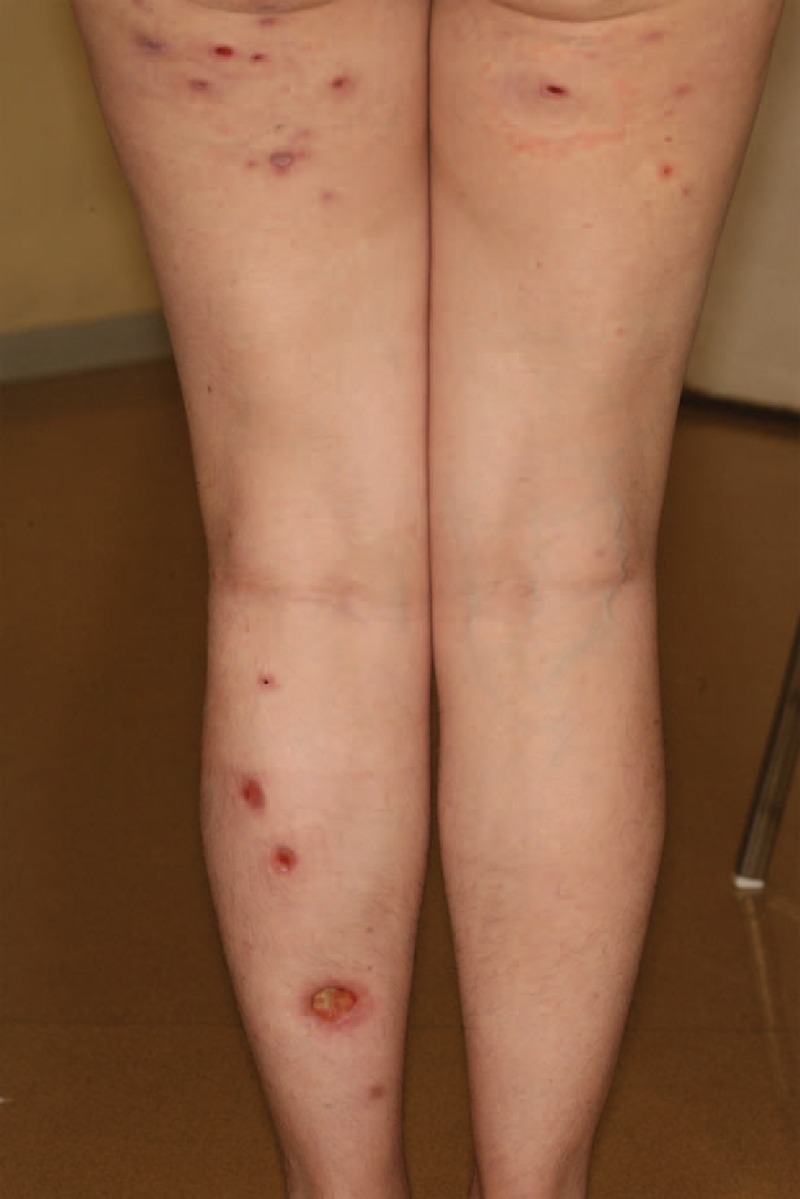
Multiple pyoderma gangrenosum lesions in the context of systemic LE (case 1). LE = lupus erythematosus.

**FIGURE 3:**
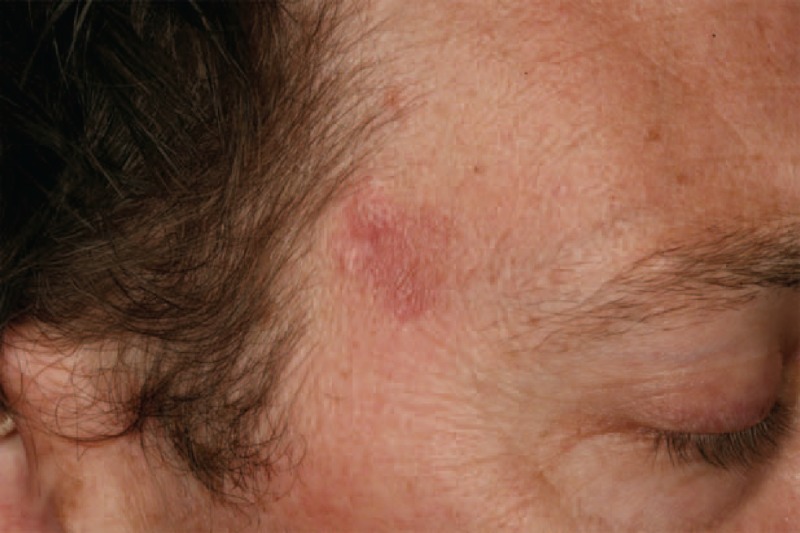
Sweet-like ND of the face in the context of subacute cutaneous LE (case 2). LE = lupus erythematosus, ND = neutrophilic dermatosis.

**FIGURE 4:**
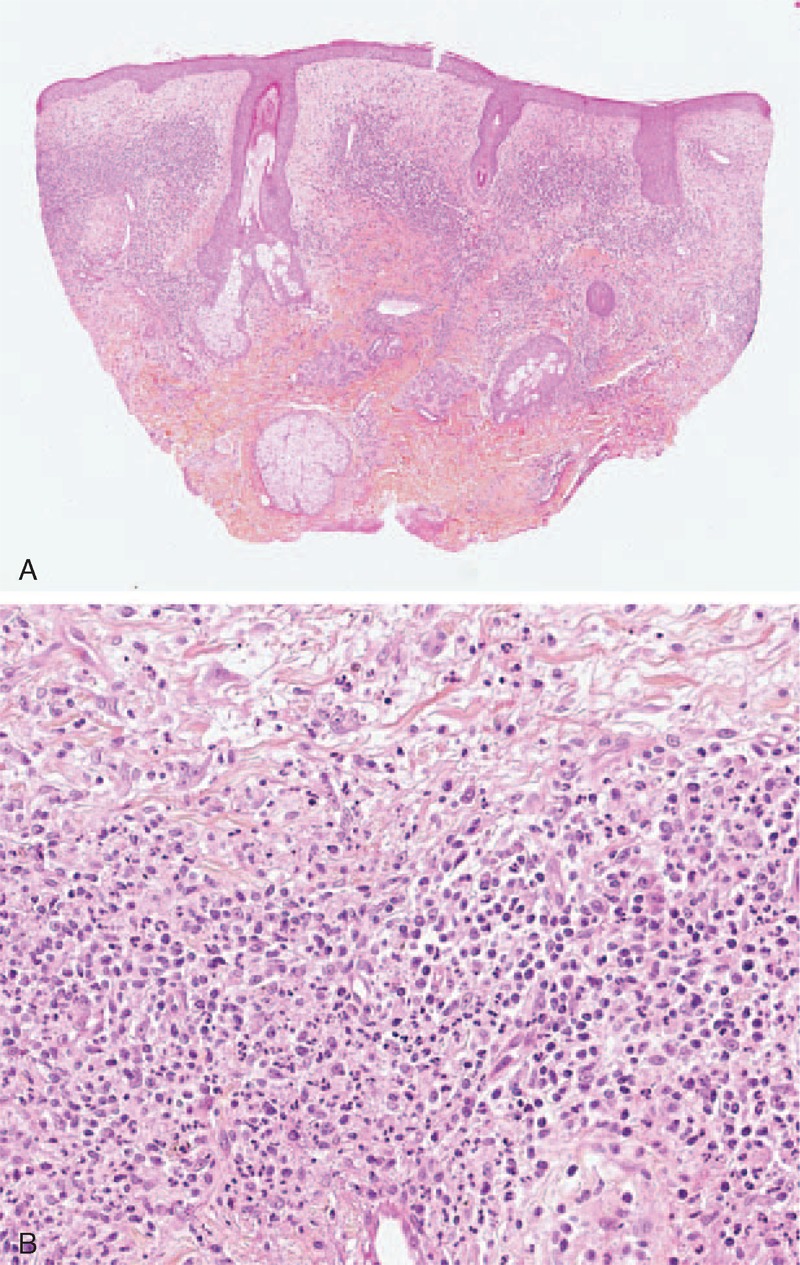
(A) Dense dermal polymorphic infiltrate composed primarily of neutrophils suggestive of Sweet syndrome (case 2) (hematoxylin–eosin stain; original magnifications, 20). (B) Dense dermal infiltrate primarily neutrophilic with edema suggestive of Sweet syndrome (case 2) (hematoxylin–eosin stain; original magnifications, 200).

**FIGURE 5:**
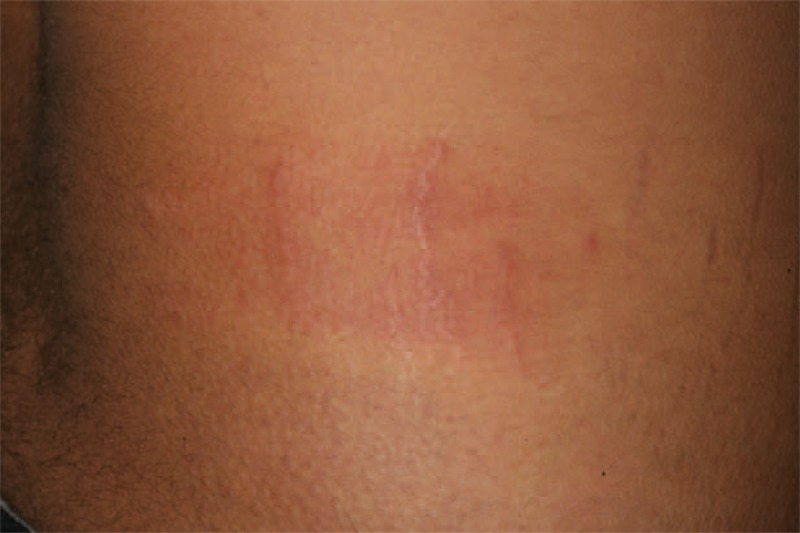
Neutrophilic urticarial dermatosis on white striae in the context systemic LE (case 3). LE = lupus erythematosus.

**FIGURE 6:**
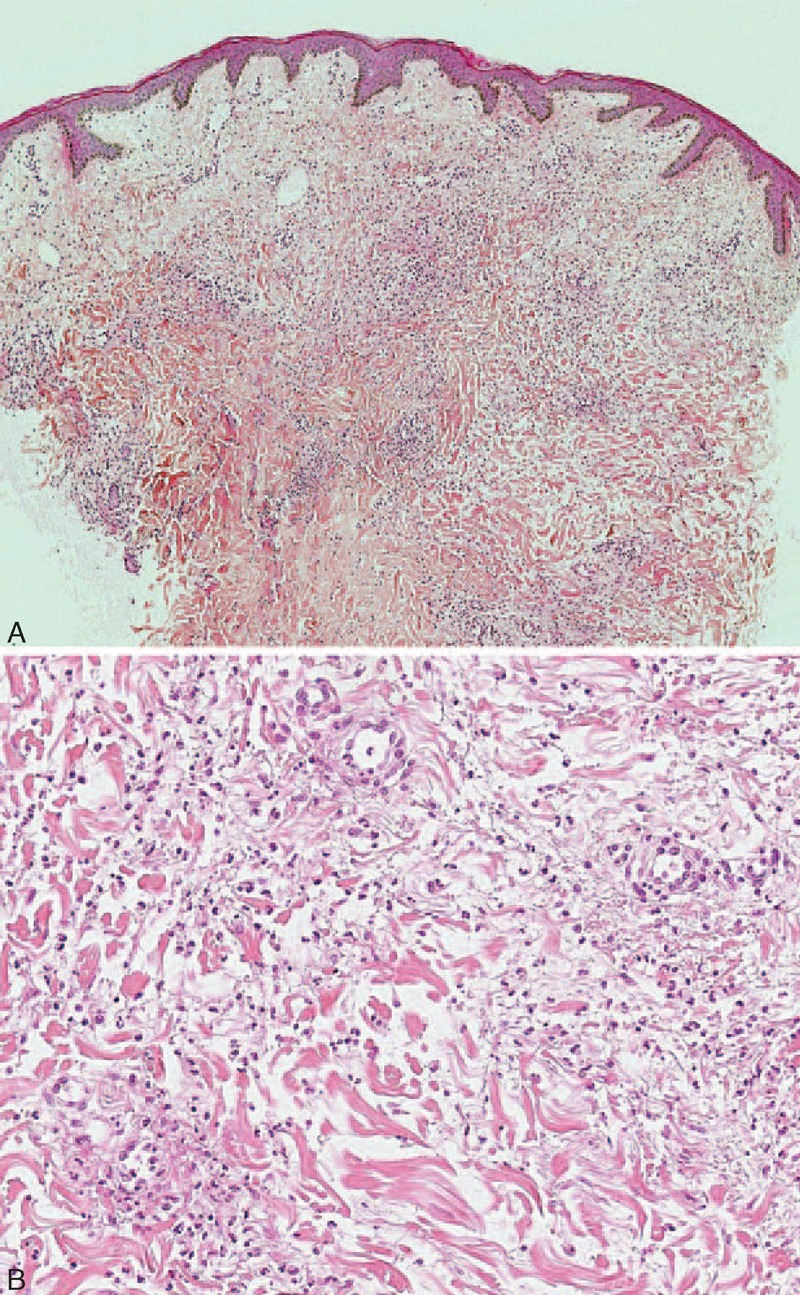
(A) Interstitial diffuse infiltrate of neutrophils associated with leukocytoclastic debris, without vasculitis suggestive of neutrophilic urticarial dermatosis (case 3) (hematoxylin–eosin stain; original magnifications, 40). (B) Intradermal diffuse neutrophilic infiltrate (case 3) (hematoxylin–eosin stain; original magnifications, 200).

**FIGURE 7:**
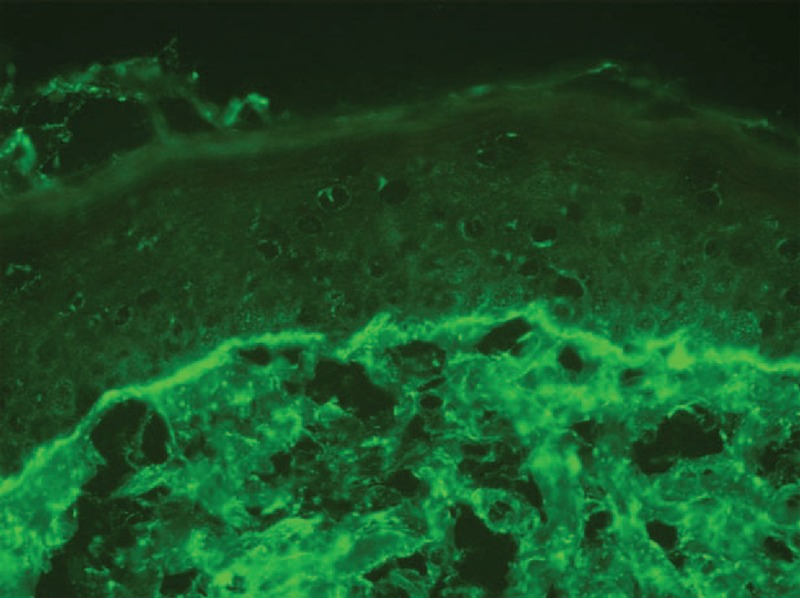
Immunofluorescence showed granular deposition of IgG at the dermal–epidermal junction (case 5).

**FIGURE 8:**
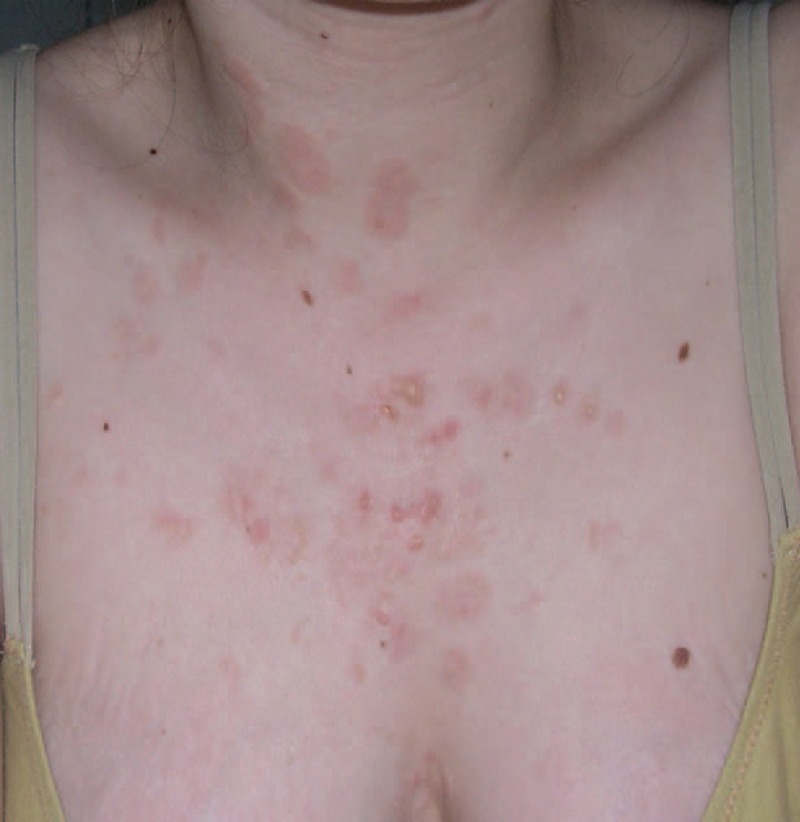
Bullous LE on sun-exposed areas (case 6). LE = lupus erythematosus.

**FIGURE 9:**
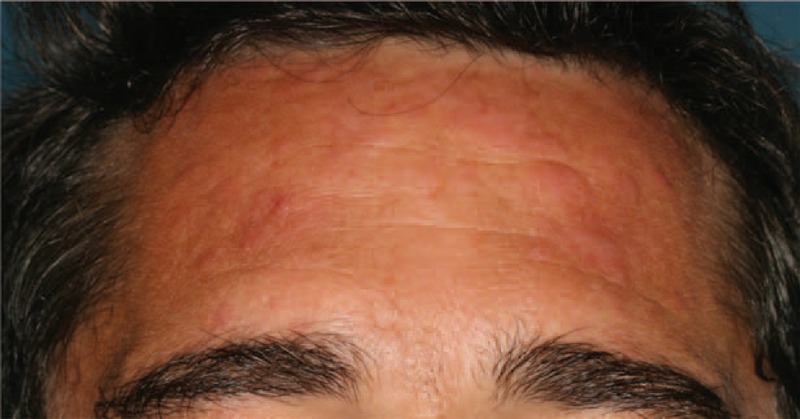
Autoimmunity-related ND in the context of cutaneous LE tumidus (case 7). LE = lupus erythematosus, ND = neutrophilic dermatosis.

**FIGURE 10:**
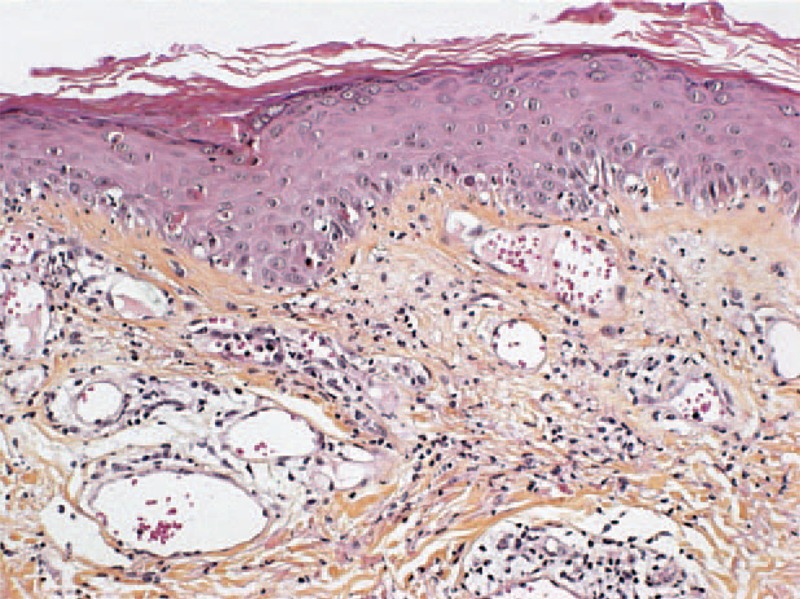
Interface dermatitis associated with slight infiltrate composed primarily with neutrophils and few lymphocytes, suggestive of autoimmunity-related ND (case 8) (hematoxylin–eosin stain; original magnifications, 200). ND = neutrophilic dermatosis.

**FIGURE 11:**
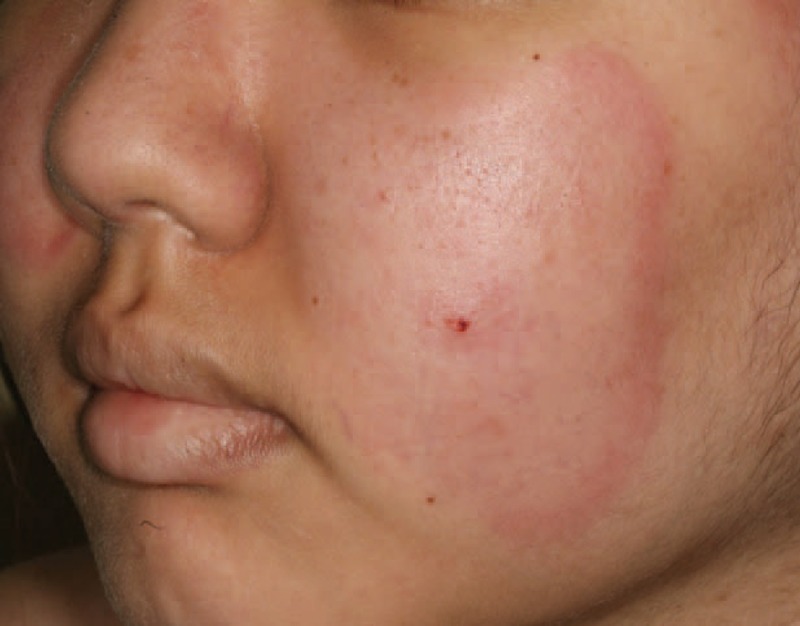
Neutrophilic annular erythema of the cheek in the context of Sjögren syndrome (case 9).

A total of 9 patients fulfilled both the histopathological and clinical criteria. A summary of clinical signs and laboratory tests of cases is depicted in Table 1. Pictures of clinical and histological (hematoxylin and eosin stain or direct immunofluorescence, original magnification ×20, ×40, or ×200) skin lesions are shown in Figures 2–11. We also performed a literature review of AICTD-associated NDs cases and discussed the possible pathophysiological link regarding this association. We distinguished, within our cases and literature ND cases, a large clinical and histological spectrum of neutrophilic skin lesions associated with AICTDs. Some of the NDs are likely to occur in both autoinflammatory and autoimmune diseases, such as pyoderma gangrenosum; other NDs seem to be specifically encountered in the setting of autoimmunity, such as bullous LE or amicrobial pustulosis of folds. However, the fact that some of the NDs have not been reported in the context of autoinflammatory syndromes does not mean that they do not have an underlying autoinflammatory mechanism. As described below, a better terminology may be required for describing AICTD-associated NDs: Sweet syndrome, Sweet-like ND, and neutrophilic urticarial dermatosis do not always have well-defined boundaries; historically, the term “Nonbullous neutrophilic lupus erythematosus” includes various entities and may be inadequate.^21^

**TABLE 1:**
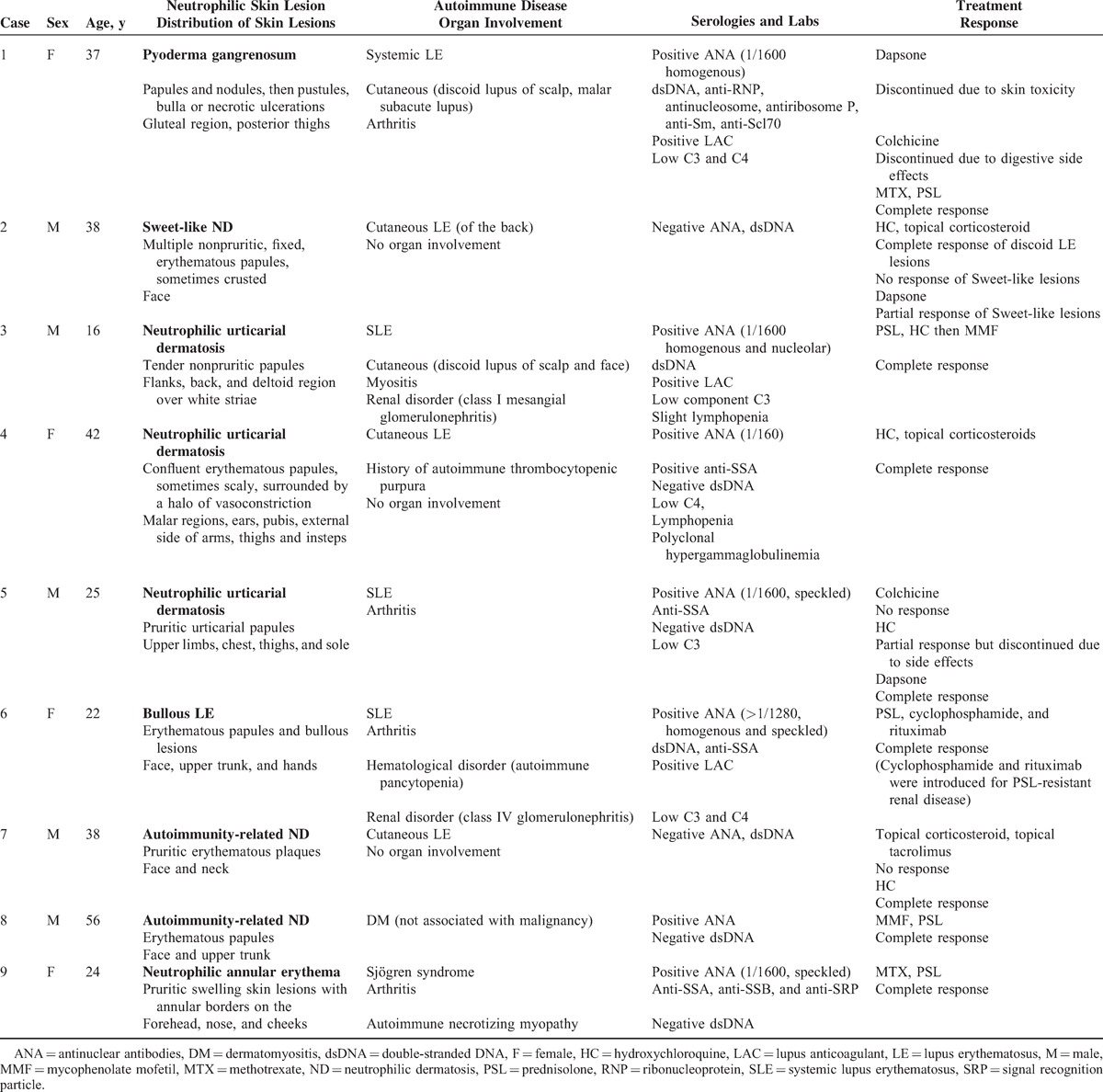
Summary of Clinical Signs and Laboratory Tests of Cases

### Pyoderma Gangrenosum and SLE

Pyoderma gangrenosum is characterized by painful nodular, bullous, or pustular lesions, which eventually ulcerate. There are no specific histological findings or pathognomonic laboratory tests for diagnosis of pyoderma gangrenosum. Of monogenic autoinflammatory diseases, pyoderma gangrenosum is associated with an underlying systemic disease in 50% to 70% of cases.^26^ The underlying diseases primarily include inflammatory bowel diseases, arthritis, IgA monoclonal gammopathies, and myeloid hematological malignancies; however, pyoderma gangrenosum may also occur on its own. Rheumatoid arthritis is the most common AICTD reported in association with pyoderma gangrenosum (10% of cases, in a review of 348 patients afflicted with pyoderma gangrenosum).^27^ SLE and pyoderma gangrenosum is an uncommon association. Seventeen cases of pyoderma gangrenosum associated with SLE,^9,28–41^ 3 cases associated with Sjögren syndrome,^42–44^ and 1 case associated with dermatomyositis^45^ have been reported in the literature. As in our case (case 1, Table 1 and Figure 2), in patients with SLE-associated pyoderma gangrenosum, the occurrence of pyoderma gangrenosum and the response to treatment were correlated with the SLE activity. Similar lesions to pyoderma gangrenosum have also been described in patients with SLE-associated antiphospholipid syndrome.^9^

### Sweet Syndrome and AICTDs

The diagnostic criteria of Sweet syndrome must include both major and 2/4 minor criteria. The 2 major criteria are erythematoedematous plaques or nodules and a dense neutrophilic infiltrate without evidence of primary vasculitis, on biopsy. The minor criteria include the following: excellent response to steroid treatment; periods of fever or malaise; preceding nonspecific respiratory or gastrointestinal infection, vaccination, hematoproliferative disorder, solid tumor, pregnancy, or autoimmune disease; increased erythrocyte sedimentation rate.^46^ Most Sweet syndrome cases are considered to be idiopathic, but Sweet syndrome can be associated with hematological malignancy, solid tumors, and drugs.^47^ Sweet syndrome also has an association with inflammatory bowel disease (both Crohn disease and ulcerative colitis).^1^ Among AICTDs, Sweet syndrome has been described in association with rheumatoid arthritis (n = 9),^11,48–51^ Sjögren syndrome (n = 9),^52–57^ including a case of coexisting Sjögren syndrome and Crohn disease,^57^ DM (n = 1),^12^ undifferentiated AICTD (n = 1),^58^ and mixed AICTD (n = 1).^59^ The association of Sweet syndrome with SLE is most commonly reported; it has been reported in 28 cases, including 6 drug-induced cases (hydralazine, n = 4^60–63^; acyclovir, n = 2^64,65^) and 22 cases that often occurred simultaneously with the onset of LE.^10,21,51,58,66–78^ The skin lesion features may not accurately meet the Sweet syndrome diagnostic criteria; in these cases, a diagnosis of Sweet-like ND has been proposed in the literature (see below).

### Sweet-Like ND and AICTDs

In daily care, as in literature case reports, the difference between Sweet syndrome and Sweet-like ND is not clear. Sweet-like ND is defined as a neutrophil-predominant infiltrate of the dermis with leukocytoclasia and superficial dermal edema. Sweet-like ND should be distinguished from Sweet syndrome because of its subacute or chronic evolution, its occurrence on sun-exposed sites, and the absence of fever or malaise. Unlike typical Sweet syndrome, skin biopsy may also show a moderate neutrophilic infiltrate and a polymorphous leukocytic and lymphocytic infiltrate. The difference between Sweet-like ND and neutrophilic urticarial dermatosis is based on the clinical aspect (macules in neutrophilic urticarial dermatosis vs papules in Sweet-like ND) and the histology (more neutrophilic dermal infiltrates and dermal edema in Sweet-like ND than in neutrophilic urticarial dermatosis).^14^ Case 2 (Table 1) had both clinical (Figure 3) and histological (Figure 4A and B) features of Sweet-like ND on the face, whereas demonstrating typical discoid LE on the back. Larson and Granter^79^ reported 14 cases of “Systemic lupus erythematosus–associated neutrophilic dermatosis,” including 6 cases that match the Sweet-like ND description. Together, we found 12 cases of Sweet-like ND associated with LE (case 2 and references^13,79^). It is likely that Sweet syndrome and Sweet-like ND associated with AICTDs belong to the same entity.

### Histiocytoid Sweet Syndrome

Camarillo et al^15^ described 2 cases of pediatric patients that presented with asymptomatic erythematous and/or violaceous papules, plaques, nodules, and papulovesicles, affecting the extremities, trunk, and face in the setting of SLE or cutaneous LE. Histopathological findings showed an infiltrate of histiocytoid myeloid cells, confirmed by immunostaining (CD68 and myeloperoxidase), accompanying neutrophils, nuclear dust, and leukocytoclastic debris. Camarillo et al coined these lesions “nonbullous histiocytoid neutrophilic dermatitis.” “Nonbullous histiocytoid neutrophilic dermatitis” is distinct from histiocytoid Sweet syndrome because it is associated with AICTDs but not with malignant neoplasms,^80^ fever and general symptoms are absent,^15^ and a more abundant neutrophil infiltrate is present.^15^

### Neutrophilic Urticarial Dermatosis and LE

Kieffer et al^14^ proposed neutrophilic urticarial dermatosis as a distinct entity from the neutrophilic variant of common urticaria. Neutrophilic urticarial dermatosis is characterized by an urticarial eruption: pale, flat or only slightly raised, nonpruritic macules, papules, or plaques, which disappear within hours without leaving any sequelae. Neutrophilic urticarial dermatosis has a different histological pattern than urticaria and a significant interstitial distribution of the neutrophilic infiltrate; the distribution is along the collagen bundles and in the deep part of the reticular dermis, with significant leukocytoclasia. There is usually moderate or no edema, and it is usually diffuse in neutrophilic urticarial dermatosis. Eosinophils and mononuclear cells are absent or scarce. Kieffer et al reviewed the literature on neutrophilic urticarial and identified 50 probable cases of neutrophilic urticarial dermatosis; they reported a series of 9 patients with neutrophilic urticarial dermatosis. Seven of the 9 patients, and the majority of cases of the literature, had systemic involvement, which included autoinflammatory diseases associated with NLRP3 mutations (n = 22), Schnitzler syndrome (n = 17), adult-onset Still disease (n = 5), and LE (n = 3). Schnitzler syndrome and adult-onset Still disease are multifactorial diseases that most likely involve autoinflammatory pathways. Kieffer et al speculated that many of the diseases associated with neutrophilic urticarial dermatosis are related to a disorder of the innate immunity, which eventually results in autoinflammation. In our study, cases 3, 4, and 5 had clinical and histopathological features of neutrophilic urticarial dermatosis. In contrast to the cases of neutrophilic urticarial dermatosis associated with SLE reported by Kieffer et al, which had a known diagnosis of SLE, our 3 cases occurred within the early and nonestablished stages of SLE (case 3 and 5) or cutaneous LE (case 4) (Table 1, Figures 5 and 6A and B). Direct immunofluorescence performed on the skin biopsy of case 2 showed a lupus band within the neutrophilic urticarial dermatosis aspect (Figure 7). Our cases of neutrophilic urticarial dermatosis associated with cutaneous LE may support the hypothesis that pathogenesis of early cutaneous lesions of LE may involve the innate immune system.^81^

### Palisaded Neutrophilic Granulomatous Dermatitis and AICTD

Palisaded neutrophilic granulomatous dermatitis is a rare cutaneous manifestation, which is most commonly reported with rheumatoid arthritis;^82^ however, it has also been associated with SLE (n = 12),^16,83–89^ inflammatory bowel disease, lymphoproliferative disorders, and systemic sclerosis.^90^ The clinical manifestations of palisaded neutrophilic granulomatous dermatitis include asymptomatic or intensely painful papules, nodules, linear subcutaneous indurated cordlike bands, and plaques on multiple body sites. The histological examination typically shows a dense neutrophilic infiltrate with degenerated collagen, leukocytoclastic debris, and palisading granulomas, without vasculitis. It has been proposed that the histological appearances of palisaded neutrophilic granulomatous dermatitis vary from early (dense inflammatory infiltrates, composed of lymphocytes, histiocytes, eosinophils, and neutrophils) to late stages (palisading granulomas with fibrosis) of the disease.^83,84^

### Bullous LE

Bullous LE typically affects young adults. Clinical manifestations include vesicles and bullae of acute onset, which arise from sun-exposed sites but may also be widespread. Histological findings show subepidermal vesicles-containing neutrophils with microabscesses, nuclear ‘dust,’ and fibrin at the tips of dermal papillae. Direct immunofluorescence shows linear deposition of IgA, IgG, and IgM and, to a lesser extent, C3 at the basement membrane. Indirect immunofluorescence may show antitype VII collagen antibodies (Abs), which are the same Abs found in epidermolysis bullosa acquisita.^91^ Approximately, 70 cases of bullous LE have been reported in the literature.^17^ Our case of bullous LE (case 6, Table 1 and Figure 8) displayed a particularly abundant neutrophilic skin infiltrate. As in our case, glomerulonephritis, hypocomplementemia, and antidouble-stranded DNA (dsDNA) Abs are common. Despite its neutrophilic infiltrate, classifying bullous LE as a “neutrophilic cutaneous LE”^22^ or a “ND associated with SLE” should be questioned, as bullous LE shares many clinical, histological, and immunological features with epidermolysis bullosa acquisita. Furthermore, typically acquired autoAbs-mediated blistering diseases can coexist with SLE (eg, epidermolysis bullosa acquisita,^92,93^ bullous pemphigoid,^94^ pemphigus,^95^ dermatitis herpetiformis,^96^ and linear IgA bullous dermatosis^97^).

### Amicrobial Pustulosis of the Folds and SLE

Amicrobial pustulosis of the folds has been considered to belong to the spectrum of noninfectious NDs associated with autoimmune disorders. Amicrobial pustulosis of the folds presents as small pustules that predominantly affect the face, scalp, and flexures;^19^ it may involve extracutaneous localizations, such as colonic neutrophilic ulcerations.^98^ Histopathological examination demonstrates subcorneal multilocular pustules, associated with a superficial perivascular and interstitial neutrophilic inflammatory infiltrate. Amicrobial pustulosis of the folds has been primarily described in association with SLE (n = 15), LE (n = 3), Sharp syndrome (n = 2), mixed CTDs (n = 1), Sjögren syndrome (n = 1), rheumatoid arthritis (n = 1), and organ-specific autoimmune diseases, such as autoimmune hepatitis,^99^ celiac disease, myasthenia gravis, and idiopathic thrombocytopenic purpura.^100^ In their case reports and reviews in 2007, Marzano et al^101^ showed that there were various autoAbs found in the context of amicrobial pustulosis of folds (antinuclear 76%; anti-dsDNA 24%; anti-Ro/SSA 19%; anti-ribonucleoprotein 14%; antismooth-muscle 10%; others ≤5%). Skin flares of amicrobial pustulosis of folds were not associated with systemic autoimmune disease flares. Lee et al^102^ reported the only case of amicrobial pustulosis of folds occurring in the context of Crohn disease, which is considered as a polygenic autoinflammatory syndrome. The pustular lesions, which were essentially located on the scalp of a 22-year-old woman, occurred under long-term therapy with infliximab; this may be considered as a cutaneous complication of antitumor necrosis factor α (TNFα) treatment. Moreover, interface dermatitis on histological examination was suggestive of induced cutaneous LE.^102^

### Autoimmunity-Related ND

Saeb-Lima et al^20^ used the term autoimmunity-related ND to describe an entity of specific AICTD lesions (urticarial or erythematous to violaceous papules, plaques or nodules, with histological features of interstitial and perivascular neutrophilic infiltrate with leukocytoclasia, vacuolar alteration along the dermal–epidermal junction, and no vasculitis) with unusual neutrophilic infiltrate, which were frequently, but not exclusively, encountered in the setting of SLE (also encountered in association with rheumatoid arthritis and Sjögren syndrome).^20^ In 2006, Gleason et al^21^ used the term “Nonbullous neutrophilic lupus erythematosus” to describe the same entity exclusively encountered in the setting of SLE; this was followed by the description by Brinster et al^103^ in 2012.

#### Autoimmunity-Related ND and LE

We described 1 case (case 7, Table 1 and Figure 9) of cutaneous lesions of LE with classical histological features of LE, that is, vacuolar alteration of the basal cell layer of the epidermis, a patchy dermal lymphocytic infiltrate (interface dermatitis) associated with an unusual neutrophilic infiltrate. We found 20 cases in the literature, with similar histological features.^13,20,21,79,103,104^ The clinical presentation of skin lesions was either “classical” LE (erythematous annular or discoid macules or plaques, n = 9)^79,104^ or “non-classical” LE (erythematous macules, urticarial papules, or plaques, n = 11).^13,20,21,103^

#### Autoimmunity-Related ND and DM

We described 1 DM patient (case 8, Table 1) in whom a skin biopsy showed histological classical features of DM, that is, interface dermatitis and edema but with a significant neutrophilic infiltrate (Figure 10). Generally, DM skin infiltrate is rare and mainly includes CD4^+^ T lymphocytes, some macrophages, plasmacytoid dendritic cells, histiocytes, plasma cells, and eosinophils.^26^ Ito et al^105^ reported a case of specific DM skin lesions on the back of the hands, face, and trunk; a skin biopsy showed a diffuse infiltration of neutrophils through the dermis, with a few lymphocytes. Caproni et al^106^ studied the immunophenotype of cells infiltrating the skin pathognomonic lesions (Gottron papules or Gottron sign) of 8 patients with DM. They showed that the main infiltrating cells were activated CD4^+^ T cells. The large quantity of myeloperoxidase-positive cells led to the conclusion that neutrophil granulocytes were the second most abundant population; these cells infiltrated the perivascular upper dermis and, oftentimes, the epidermis.

### Neutrophilic Annular Erythema and Sjögren Syndrome

Clinical characteristics of annular erythema include an elevated erythematous border and central pallor, suggestive of Sweet syndrome; a red scaly polycyclic lesion, suggestive of subacute LE; or a papulous annular erythema,^107^ without a subsequent scar or pigmentation.^108^ Histological features of annular erythema include a deep perivascular and/or periappendageal polymorphic infiltrate, primarily composed of lymphocytes, which may be associated with neutrophils and plasma cells.^108^ First described in a Japanese series of 22 cases in 1989,^108^ the association of annular erythema and Sjögren syndrome has mostly been reported in the Asian population; annular erythema is more often associated with cutaneous LE in the occidental population.^109^ Although most published cases show mixed lymphocytic infiltrate with some neutrophils,^107^ there have been few reported cases of neutrophilic infiltrate histologically.^110–116^ We report the first case of annular erythema of the face with significant neutrophilic infiltrate, associated with AICTD (case 9, Table 1 and Figure 11). We chose to designate this case with the term “neutrophilic annular erythema” because clinical and histological signs were suggestive of Sjögren annular erythema; however, there was an unusual neutrophilic infiltrate. Notably, this case (case 9) is interesting because it describes the second^117^ reported association between necrotizing autoimmune myopathy and Sjögren syndrome. Necrotizing autoimmune myopathy is distinct from other inflammatory myopathies due to muscle necrosis and regeneration without inflammatory infiltrate on muscle biopsy; in addition, there is positivity of the antisignal recognition particle Abs.^118^

## PATHOPHYSIOLOGY

### Mechanism of Neutrophilic Infiltrate in AICTD Skin Lesions

Marzano et al studied the cytokine expression in skin lesions of pyoderma gangrenosum and Sweet syndrome as compared with healthy skin.^119^ They showed that IL-1β and its receptors, IL-8 and IL-17, TNFα, the chemokines CXCL1/2/3 and CXCL16, and metalloproteinases 2 and 9 (MMP-2, MMP-9) were significantly overexpressed in skin lesions when compared with normal skin. All of these overexpressed cytokines amplify the inflammatory response and neutrophil recruitment. The pathogenesis of pyoderma gangrenosum in inflammatory bowel disease has also been proposed to be an abnormal immune response consisting of cross-reacting Abs directed towards common antigens in the bowel and skin.^120^ The neutrophilic infiltrate in AICTDs may also be explained by the activation of the innate immune system because of immune complex deposition and complement cascade activation; it may also be due to a global aberrant innate immune response to environmental triggers. The adaptive immune system may also be involved in the neutrophilic infiltration, particularly through the T helper lymphocytes (Th) 17-cell subset. Th17 cells secrete neutrophil-recruiting chemokines, such as IL-17A and G-CSF. Local synthesis of IL-17A by infiltrating Th17 cells in skin lesions of AICTDs could explain the presence of a nonspecific neutrophilic infiltrate in specific skin lesion of AICTDs, which are classified as autoimmunity-related ND. Neutrophils may also be overrecruited in AICTD skin lesions because of an abnormal expression and/or activation of adhesion/migration molecules through the endothelial barrier of inflamed tissues.^121^ Caproni et al^106^ proposed that the neutrophilic infiltrate described in specific skin lesions of DM could be explained by increased expression of adhesion molecules, such as intercellular adhesion molecule-1, vascular cell adhesion molecule-1, and E-selectins on endothelial cells.

### Pathogenic Role of the Neutrophilic Infiltrate in AICTD Skin Lesions

Aberrant and/or excessive neutrophil extracellular trap (NET) formation (NETosis) is increasingly believed to play an important role in the development and perpetuation of autoimmune diseases. NETs describe nuclear chromatin fibers actively extruded from the neutrophil in response to stimulating signals (lipopolysaccharide, IL-8, TNFα, bacteria, fungi, and parasites).^122^ NETs bind histones, IL-17, and antimicrobial components to kill invading microbes. In peripheral blood of SLE patients, an abnormal neutrophil subset (the low density granulocytes) has an increased capacity to synthesize NETs. Affected skin and kidneys from LE patients are infiltrated by these netting neutrophils.^123^ In addition, sera from patients with active SLE have a reduced ability to degrade in vitro generated NETs.^124,125^ Antimicrobial components exposed by NET drive an immunostimulatory signal, which facilitates the uptake and recognition of self-dsDNA, histones, and IFNα synthesis by plasmacytoid dendritic cells.^122,123^ This process could induce the activation of autoimmune T and B lymphocytes. Activated autoreactive lymphocytes induce DNA-containing immune complexes and lead to IL-17 production; this may trigger neutrophil activation and NET formation,^121^ leading to a vicious cycle.

## THERAPEUTIC

The treatment strategies of NDs consist of modulating the neutrophilic activation, maturation, or migration. Oral corticosteroid use is efficient and has been proven be effective. Sulphones (dapsone), colchicine, potassium iodide, retinoid (acitretin), clofazimine, sulfasalazine, and thalidomide may also be beneficial. Severe corticosteroid-unresponsive cases may be treated with immunosuppressant agents, such as cyclosporine, cyclophosphamide, chlorambucil, intravenous tacrolimus, and mycophenolate mofetil. Recently, TNFα inhibitors (infliximab, adalimumab, and etanercept) have shown good efficacy in pyoderma gangrenosum, especially when pyoderma gangrenosum was associated with inflammatory bowel diseases; this therapy concurrently cured both disease processes.^126^ In our study, 2 cases (cases 2 and 5) were improved by neutrophil targeting therapy, that is, dapsone; underlying AICTD therapies, that is, prednisolone, hydroxychloroquine, methotrexate, mycophenolate mofetil, and cyclophosphamide, were effective on neutrophilic skin lesions in the remaining 7 cases, suggesting a parallelism between the 2 disorders.

## CONCLUSION

NDs in the setting of AICTDs include many entities in which autoimmune and autoinflammatory pathways are more or less interconnected, which may explain the polymorphic clinical spectrum of the neutrophilic skin symptoms. This review supports the idea that AICTD neutrophilic skin lesions are a distinct entity within the spectrum of cutaneous signs in AICTDs, as has been previously reported.^22^ Further investigations are needed to clarify the probable part of the neutrophil and innate immunity in the pathogenesis and prognosis of AICTDs; this may lead to new therapeutic targets.
